# Case Report: Adenosine kinase deficiency diagnosed 10 years after liver transplantation: Novel phenotypic insights

**DOI:** 10.3389/fped.2022.1061043

**Published:** 2022-12-14

**Authors:** Patryk Lipiński, Elżbieta Ciara, Dorota Jurkiewicz, Maciej Pronicki, Elżbieta Jurkiewicz, Anna Bogdańska, Rafał Płoski, Irena Jankowska

**Affiliations:** ^1^ Department of Pediatrics, Nutrition and Metabolic Diseases, The Children's Memorial Health Institute, Warsaw, Poland; ^2^ Department of Medical Genetics, The Children's Memorial Health Institute, Warsaw, Poland; ^3^ Department of Pathology, The Children's Memorial Health Institute, Warsaw, Poland; ^4^ Department of Diagnostic Imaging, The Children's Memorial Health Institute, Warsaw, Poland; ^5^ Department of Biochemistry, Radioimmunology and Experimental Medicine, The Children's Memorial Health Institute, Warsaw, Poland; ^6^ Department of Medical Genetics, Medical University of Warsaw, Warsaw, Poland; ^7^ Department of Gastroenterology, Hepatology, Nutritional Disorders and Paediatrics, Children's Memorial Health Institute, Warsaw, Poland

**Keywords:** adenosine kinase, adenosine kinase deficiency, liver transplantation, S-adenosyl homocysteine, S-adenosylmethionine

## Abstract

Adenosine kinase (ADK) deficiency is a rare inborn error of methionine and adenosine metabolism. So far, a total of 27 patients with ADK deficiency have been reported. Here, we describe the first Polish patient diagnosed with ADK deficiency, aiming to highlight the clinical presentation of disease, emphasize diagnostic difficulties, and report the long-term follow-up. Six-month-old patient presented with cholestatic liver disease, macrocytic anemia, developmental delay, generalized hypotonia, delayed brain myelination, and elevated levels of serum methionine. A decrease of mitochondrial respiratory chain complex II and III activity were found in the postnuclear supernatants obtained from skeletal muscle biopsy. The patient underwent living-donor liver transplantation (LTx) at 14 months of age. Ten-year follow-up after LTx revealed a preserved good liver function, persistent regenerative macrocytic anemia, progressive neurological disease but disappearance of brain MR changes, short stature, and cortisol deficiency. Whole exome sequencing revealed the patient to be affected with two novel *ADK* variants, which pathogenicity was confirmed biochemically by demonstration of elevated concentration of S-adenosylhomocysteine.

## Introduction

Adenosine kinase (ADK) deficiency (# 614300) is a rare inborn error of methionine and adenosine metabolism (1, 2). The mechanism for disease is biallelic loss of function, with reduced ADK activity. ADK (EC 2.7.1.20) has an important role in maintaining cellular metabolism, including energy homeostasis and transmethylation pathways (1). ADK converts adenosine to adenosine monophosphate (AMP) while its deficiency leads to adenosine accumulation and reversal of S-adenosylhomocysteine hydrolase (SAHH) deficiency with elevated plasma concentrations of S-adenosylhomocysteine (SAH), S-adenosylmethionine (SAM) and methionine (1, 2).

ADK deficiency was first clinically described by Bjursell et al. in 2011 (3). So far, a total number of 27 patients with ADK deficiency have been reported (2–7). ADK-deficient patients presented with liver dysfunction (most commonly with prolonged neonatal cholestatic jaundice), delayed psychomotor development, and neurological features including generalized hypotonia and epilepsy (2–7). The final diagnosis was usually confirmed by genetic studies, including *ADK* gene sequencing or whole exome sequencing (WES).

Here, we report on the first Polish patient diagnosed with ADK deficiency based on molecular and biochemical analyses. We would like to highlight the manifestation of disease as cholestatic liver cirrhosis requiring liver transplantation (LTx), emphasize the diagnostic difficulties with depicting the role of genetic studies, and report the long-term follow-up.

## Case presentation

**Figure 1:**
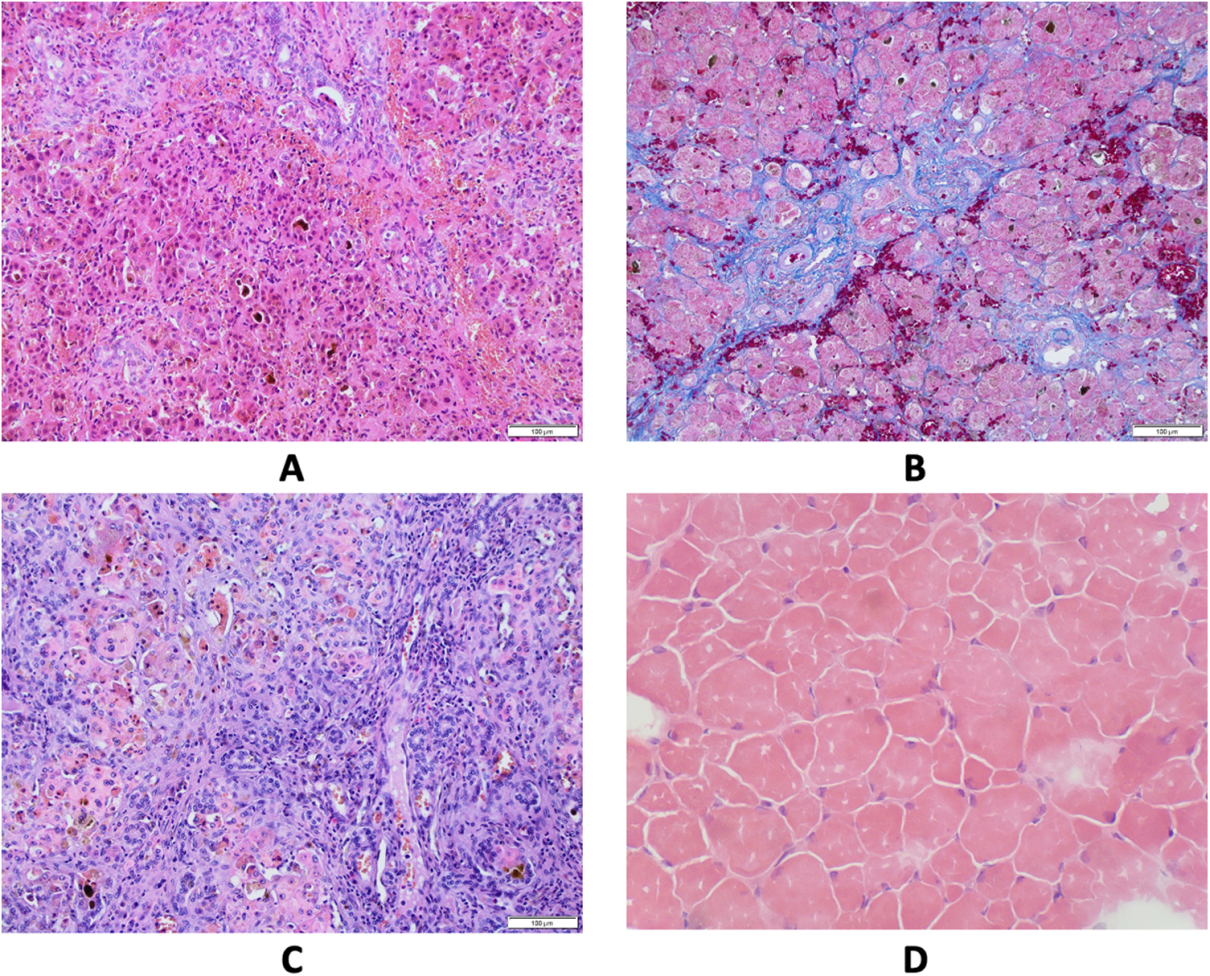
Results of pathological studies. (**A**) Liver core biopsy at the age 4 months showing mainly periportal fibrosis and cholestasis. Hematoxylin and eosin stain, original magnification 200×. (**B**) Liver core biopsy at the age 7 months revealed progression of periportal fibrosis toward perisinusoidal pattern. AZAN stain, original magnification 200×. (**C**) Explanted liver sample at cirrhotic stage. Hematoxylin and eosin stain, original magnification 200×. (**D**) Skeletal muscle biopsy showing variability of muscle fiber diameters. Hematoxylin and eosin stain, frozen section, original magnification 200×.

The patient was the first child of non-consanguineous Polish parents born from an uneventful pregnancy at 41 weeks of gestation with a birth mass of 3.600 g and length of 55 cm (0.62 standard deviation score (SDS)). From the first days of life, a macrocytic anemia (requiring red blood cells concentrate infusions), cholestatic jaundice with normal serum gamma-glutamyltransferase (GGT) activity and slightly elevated serum transaminases, metabolic acidosis with slightly elevated serum lactates, and recurrent episodes of hypoglycemia were observed, see Table 1. Normal liver and spleen volume were observed. Echocardiography revealed the patent ductus arteriosus (PDA) with ventricular volume overload requiring its surgical ligation. Infectious causes of cholestasis, including hepatitis B virus (HBV), hepatitis C virus (HCV), cytomegalovirus (CMV), Epstein–Barr virus (EBV), human immunodeficiency virus (HIV), and *Toxoplasma gondii* infections were excluded serologically. Alpha-1-antitrypsin deficiency, cystic fibrosis, galactosemia, and congenital hypothyroidism were excluded as well. Urinary gas chromatography–mass spectrometry (GC/MS) analysis revealed elevated levels of lactic acid, 4-hydroxyphenylacetic acid, and 4-hydroxyphenylpyruvate acid, while succinylacetone was not detected. Isoelectric focusing of serum transferrin was normal. Tandem mass spectrometry analysis (MS/MS) of serum acylcarnitines revealed normal results while of serum amino acids (AA) revealed elevated levels of methionine (541 μmol/L, reference range 6–81 μmol/L) only. Cholescintigraphy showed no excretion of the radiotracer into the bowel; thus, intraoperative cholangiography was performed revealing normal extrahepatic bile ducts. Then, surgical liver biopsy was performed showing cholestasis and periportal fibrosis, see Figure 1A. The patient was referred to our department, which serves as a pediatric hepatology referral center.

**Table 1:** Results of laboratory analyses.

Parameter and reference values	Age									
	Second day of life	2 months	3 months	6 months	13 months	20 months	2.5 years	4 years	7 years	12 years
Hemoglobin (g/dl)	9.6	13.1	10.7	12.1	10.4	8.7	9.0	8.8	9.1	9.9
Platelets (150–450 K/μl)	289	165	350	490	130	250	300	429	320	350
Total serum bilirubin (<1.00 mg/dl)	9.8	9.9	11.0	8.6	15.2	1.1	0.54	0.57	0.61	0.57
Direct serum bilirubin (<0.2 mg/dl)	7.0	7.7	9.3	7.7	13.9	0.4	0.2	0.1	0.11	0.13
AST (<60 U/L)	87	169	131	235	200	18	42	34	25	23
ALT (<60 U/L)	192	56	115	150	102	11	43	30	29	28
GGT (<200 U/L)	45	69	114	109	199	11	55	13	25	
INR (0.9–1.3)	n.a.	1.37	1.52	1.49	1.84	1.02	1.15	1.09	1.05	1.08
Albumin (3.8–5.4 g/dl)	n.a.	n.a.	4.6	4.9	4.6	4.8	4.2	3.9	4.3	4.1
Serum bile acids (<10.0 μmol/L)	n.a.	n.a.	n.a.	149	200	<10	<10	<10	<10	<10

AST, aspartate aminotransferase; ALT, alanine aminotransferase; GGT, gamma-glutamyltransferase; INR, international normalized ratio; n.a., not analyzed.

**Figure 2:**
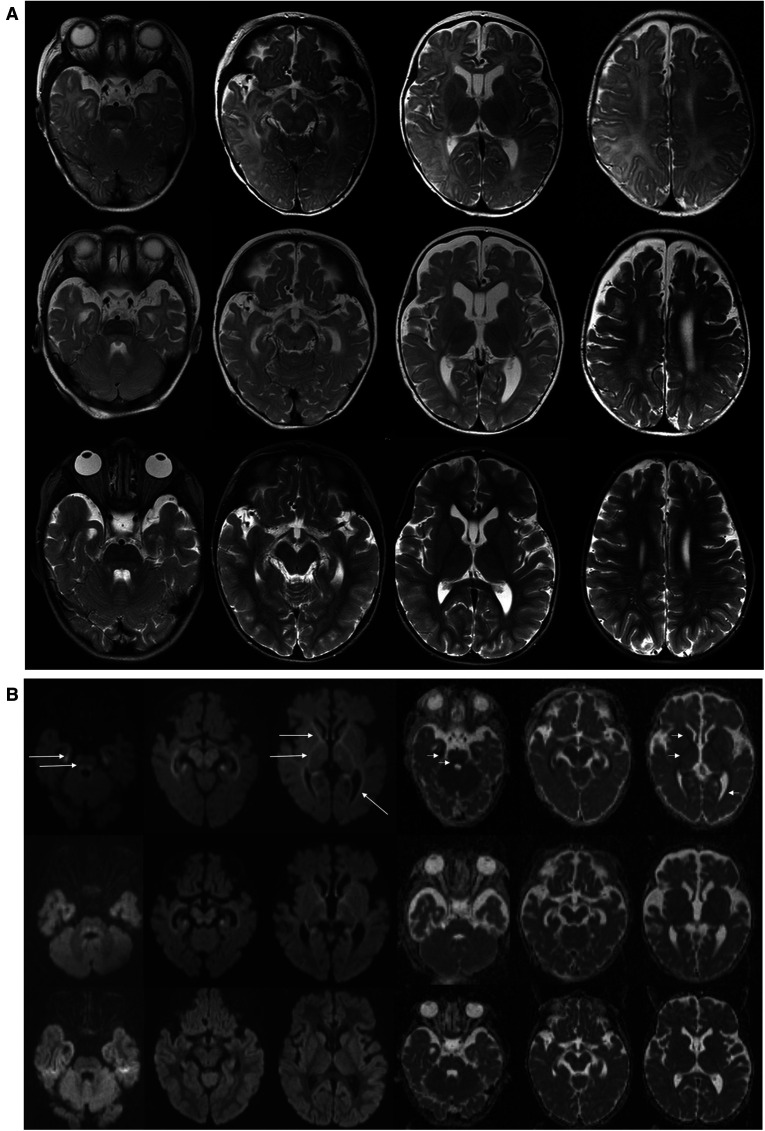
Brain MR examinations performed at 6 months (upper row), 14 months (middle row), and 5 years (bottom row). (**A**) Axial T2-WI. T2-weighted images at 6 months showed delayed brain myelination; on follow-up examination at 14 months, myelination was progressed, but it still incomplete. On last examination at 5 years, myelination was complete. Brainstem with tegmental hyperintensity was seen at 6 and 14 months and was almost completely resolved at 5 years. Width Virchow–Robin spaces appeared at 5 years. (**B**) Axial DWI (diffusion weighted images with b = 1000) and ADC maps. At 6 months, abnormal restricted diffusion (hyperintense in DWI—long arrows and hypointense in ADC maps—short arrows) involving anterior and posterior limbs of internal capsules, anterior parts of external capsules, mesial temporal white matter, optic radiations were seen. Restricted diffusion in the cerebral peduncles as well as in the central tegmental tracts and superior cerebellar peduncles are shown. At 14 months, images showed milder degree of residual restricted diffusion which returned to normal at 5 years. ADC, apparent diffusion coefficient.

On admission, at 6 months of age, the patient presented with jaundice, slightly enlarged liver (palpable 2 cm below the costal margin) and enlarged spleen (palpable 4–5 cm below the costal margin), developmental delay, and generalized hypotonia. Notably, rising parameters of cholestasis with high serum biliary acids (BA) concentration as well as still highly elevated serum transaminases were noted (Table 1). Due to the presence of neurological features, magnetic resonance (MR) imaging of the brain was performed revealing a delayed brain myelination and noncharacteristic features, see Figure 2. Metabolic work-up was then repeated. Urinary GC/MS analysis revealed again elevated levels of 4-hydroxyphenylacetic acid and 4-hydroxyphenylpyruvate acid, while succinylacetone was not detected. MS/MS analysis of serum AA revealed lower (but still elevated) levels of methionine (381 μmol/L, reference range 6–81 μmol/L) only. Based on the co-occurrence of progressive liver disease and neurological features, mitochondrial disease was suspected. For that moment, there was no possibility to perform molecular analysis. Thus, a second liver biopsy with skeletal muscle biopsy was performed. Liver biopsy revealed the progression of periportal fibrosis toward perisinusoidal pattern, see Figure 1B. Skeletal muscle biopsy showed variability of muscle fiber diameters (Figure 1C), mild lipid accumulation in sarcoplasm, and increase of subsarcolemmal activity of oxidative enzymes in muscle fibers. No other myopathic or neurogenic signs were noted. Results of mitochondrial respiratory chain (RC) complexes and citrate synthase (CS) activities are presented in Table 2. Decreased activity of RC complex II and III and increased CS activity were found. All findings were concluded as nonspecific, requiring further multidisciplinary discussion.

**Table 2:** Results of mitochondrial respiratory chain and citrate synthase activities.

RC complex and its activity	CS (nmol/min/mg protein)	Complex I (NADH—ubiquinone oxidoreductase) (% CS)	Complex II (succinate—ubiquinone oxidoreductase) (% CS)	Complex II + III (succinate—cytochrome c oxidoreductase) (% CS)	Complex III (coenzyme Q—cytochrome c oxidase) (% CS)	Complex IV (cytochrome c oxidase) (% CS)
Normal value	96.5–150.1	8.2–18.0	7.5–12.5	44.9–93.5	5.7–9.5	6.2–17.5
Patient's results	**450.9**	15.1	**7.4**	**43.9**	**4.8**	10.9

RC, respiratory chain; CS, citrate synthase.

Bold values indicate abnormal results.

Progression of liver disease was observed, see Table 1. Finally, the patient underwent AB0-incompatible living-donor LTx at 14 months of age. The patient's length at that age was 70 cm (−1.99 SDS). Liver cholestatic cirrhosis was confirmed grossly and microscopically in the explanted organ, see Figure 1D. Tacrolimus and prednisone were used for immunosuppressive treatment.

The patient was then systematically consulted during control visits at the Outpatient Hepatology Clinic. The child was able to walk independently at 5 years of age but clumsiness was observed. Control brain MR, performed at that age, showed normal results, see Figure 2. Liver function tests had normalized after LTx but the regenerative macrocytic anemia persisted (with hemoglobin concentration varying between 7.8 and 10 g/dl), see Table 1. Bone marrow myelogram revealed an extremely rich celled marrow with significantly stimulated megaloblastic erythropoiesis with suppressed granulocytic system and multiple megakaryocytes and clusters of platelets. Gastrointestinal endoscopy did not reveal any signs of bleeding; however, eosinophilic esophagitis was accidentally diagnosed at 9 years of age. Till the first years of life, the patient presented with failure to thrive. Extensive endocrinologic assessment at 10 years revealed the patient to be with a short stature (101 cm, −6.19 SDS), periodic hypoglycemia (with normal insulin levels), and cortisol deficiency. On neurological examination, the patient presented with intellectual disability, ataxia, spasticity, and peripheral neuropathy.

In 2022, 10 years after LTx, the patient underwent molecular analysis as a part of a WES study in patients with liver disease of an unknown etiology. WES analysis revealed two novel missense variants c.149T > G, p.(Leu50Arg) and c.641T > C, p.(Leu214Pro) in the *ADK* gene (NM_006721.4). The parents were identified as carriers of these variants. Both variants were predicted *in silico* as a likely pathogenic with a score of 7 according to the American College of Medical Genetics and Genomics (ACMG) classification. Subsequent biochemical analyses revealed the following: elevated serum SAM 139.5 nmol/L (reference ranges 64.1–124.9), normal serum SAH 16.3 nmol/L (reference ranges 4.9–19.7), and normal serum methionine 16.3 μmol/L (reference range 6–51). Based on the retrospective analysis of clinical presentation and current biochemical and molecular analyses, the final diagnosis of ADK deficiency was established.

## Methods

### Pathological assessment

Skeletal muscle biopsy was performed at the age of 7 months. The sample was obtained by open surgical biopsy of vastus lateralis muscle was snap frozen. Kryostat sections were stained using standard local lab panel including hematoxylin and eosin (HE); modified Gomori trichrome; Oil red O; succinate dehydrogenase; NADH dehydrogenase; cytochrome c oxidase (COX); acid phosphatase; myosin AT-ase preincubated in pH 4,3, 4,6, and 9,4; and Sirius red. Immunohistochemical studies assessing sarcolemmal expression of dystrophin, adhalin, and merosin were also included.

Liver needle core biopsy was performed at the ages of 4 and 7 months. Biopsy tissue cylinder was formalin fixed and paraffin embedded (FFPE). Paraffin sections were stained with HE, trichrome AZAN, and periodic acid-Schiff (PAS): PAS with diastase pretreatment and reticulin fiber stain. Explanted liver (LTx at the age of 14 months) was processed routinely to FFPE sections and stained with HE.

### Mitochondrial enzymes activities

Spectrophotometric assays in postnuclear supernatants obtained from skeletal muscle biopsy have been used for the measurement of complex I (NADH-ubiquinone oxidoreductase, rotenone sensitive), complex II (succinate-ubiquinone oxidoreductase, malonate sensitive), complex II + III (succinate-cytochrome c oxidoreductase, antimycin sensitive), complex IV (cytochrome c oxidase), and CS enzyme activities.

The ratio between the activity of individual respiratory chain complexes and CS was calculated to be independent of different amount/number of mitochondria in control and patient cells.

### SAM and SAH analysis

Plasma sample was acidified with formic acid and spiked with deuterated internal standard solution. Then, the sample was filtered using centrifugal filters to eliminate protein fractions. SAM and SAH were separated by high-performance liquid chromatography (HPLC) using e-gradient elution ion-pair reversed phase. Detection was carried out using a triple quadrupole mass spectrometer equipped with an electrospray ion source operating in the positive ionization mode, the Multiple Reaction Monitoring mode. The calculations of the results were performed using the Agilent Mass Hunter Workstation software according to our modification of the method described by Blau et al.

### Whole exome sequencing

Whole exome sequencing was conducted to identify the molecular basis of the disease in patient. WES was performed on a HiSeq 1500 using an Exome Enrichment Kit (Illumina) according to the published protocol (8). The identified variants were filtered according to minor allele frequency ≤ 0.001 in genomic databases (Genome Aggregation Database—gnomAD, in-house database of 8,000 exomes), conservation (Genomic Evolutionary Rate Profiling—GERP), and predicted effect on protein structure and function. *In silico* prediction tools were used for the interpretation of candidate variants, including CADD, SIFT, MutationTaster, PolyPhen2_HDIV, PolyPhen2_HVAR, Mutation Assessor, MetaSVM, MetaLR, and FATHMM. Candidate variants were classified according to the American College of Medical Genetics and Genomics and the Association for Molecular Pathology (ACMG/AMP) guidelines (9). The nomenclature of molecular variants follows the Human Genome Variation Society guidelines (HGVS) using human *ADK* reference sequence: NM_006721.4 (for cDNA) and NP_006712.2 (for protein).

## Discussion

In this study, we described a patient with ADK deficiency providing some novel phenotypic insights. For the first time, we report on the progressive course of liver disease in ADK deficiency requiring LTx. Ten-year follow-up after LTx revealed preserved good liver function, persistent regenerative macrocytic anemia, progressive neurological disease (intellectual disability, ataxia, spasticity, and peripheral neuropathy, but no epilepsy, on the contrary to other reported patients) but disappearance of brain MR changes, short stature observed till early infantile period, and cortisol deficiency diagnosed at 10 years of age. The patient was affected with two novel *ADK* variants and their pathogenicity was confirmed biochemically by demonstration of elevated concentration of SAM.

### Hepatic phenotype in ADK deficiency

Liver constitutes the highest expression levels of ADK and thus liver disease is a characteristic feature of ADK deficiency (but not mandatory) (10). Most reported patients presented with neonatal cholestatic jaundice; in most of them (12/14 according to Becker et al.) an improvement on methionine restriction diet was reported (2–7). However, single cases of spontaneous liver function improvement and resolution of cholestasis have been also reported (1, 5, 6). Histologically, hepatic steatosis, fibrosis, and cirrhosis were described in ADK-deficient patients (2–7). Becker et al. have recently reported on a patient with decompensated liver cirrhosis who died at 8 months of age (LTx was ruled out regarding the severity of neurological impairment) (6). Aside from a cholestatic pattern of liver injury, Almuhsen et al. described a patient with recurrent episodes of acute liver injury with elevation of serum transaminases and sometimes coagulopathy (7). In this study, we described an ADK-deficient patient presenting with cholestatic liver cirrhosis who required LTx. Ten-year follow-up after LTx revealed preserved good liver function. However, we would like to emphasize that LTx should not be considered in diagnosis of ADK deficiency based on the available literature, which does not support that because of limited data.

### Neurologic phenotype in ADK deficiency

Neurological manifestation, in addition to liver involvement, represents the second characteristic feature of ADK deficiency. The mechanism of brain damage is not known. However, it could be related to the lack of energy metabolites (1, 2). Adenosine is pivotal for maintenance of energy homeostasis in most organ systems including the brain and is an endogenous modulator of neuronal excitability (10, 11). Homozygous deletion of the *ADK* gene in mice resulted in the development of seizures and cognitive deficits (11). Biochemical studies revealed enhanced adenosine levels around synapses and adenosine A_2A_ receptor-dependent synaptic plasticity (11). Most ADK-deficient patients (15/27 according to Becker et al.) developed epilepsy early in life (below 2 years of age), and in some of them a refractory epilepsy finally developed (6). Conversely, our patient did not present with epilepsy during 12 years of life, and thus we could conclude that epilepsy could be a variable feature of ADK deficiency.

Psychomotor delay and muscle hypotonia were commonly reported among ADK-deficient patients and were observed in our patient (2–7). Ten-year post-LTx follow-up revealed a progressive neurological disease as a global developmental delay, ataxia, and spasticity development. Brain MR changes observed in our patient were similar to those reported in the literature (12). A delayed, but ultimately completed, brain maturation with nonspecific white matter changes and transient central tegmental tract hyperintensity were observed.

### Biochemical and molecular phenotype of ADK deficiency

Based on our patient's report, we would like to emphasize some difficulties in establishing the diagnosis of ADK deficiency. One of the main diagnostic pitfalls was that elevated levels of methionine observed in our patient were mistakenly considered as secondary to cholestatic liver disorder. Methionine is an essential amino acid that is metabolized mainly by the liver where it is converted to SAM (13). It is a well-known phenomenon that (transient) elevation of methionine serum concentration could be observed secondary to liver disease while hypermethioninemia is a biochemical hallmark of some inborn errors of metabolism, including methionine adenosyltransferase I/III deficiency, glycine-N-methyltransferase (GNMT) deficiency, S-adenosylhomocysteine hydrolase (SAH hydrolase, AHCY) deficiency, cystathionine beta-synthase deficiency, fumarylacetoacetate hydrolase deficiency, and citrin deficiency (2, 3). A recently published retrospective analysis on ADK-deficient patients performed by Becker et al. showed that methionine concentration above twofold the upper limit of normal should lead to the suspicion of ADK deficiency (6). Our case confirms this thesis. However, based on the other reported cases, we also know that methionine concentrations fluctuate and could be normal.

Regarding other biochemical markers of ADK deficiency, plasma SAH and SAM concentrations are the most reliable markers (2–7). In all the reported ADK-deficient patients, SAM was elevated while SAH was elevated in all beyond one patient described by Bjursell et al. (3). In our patient, methionine and SAH were normal while SAM was found elevated (during the last follow-up visit). This phenomenon could be related to the fact that the patient was transplanted for 10 years. Conversely, when the hepatic content of methionine is high, methionine is converted to SAH and then to homocysteine by AHCY (13). However, in a cohort of 11 patients with ADK deficiency described by Staufner et al., SAH and SAM concentrations in plasma were found elevated, even when methionine concentrations were normal (1).

Adenosine represents another potential biomarker in ADK deficiency (2–7). It was found elevated in dried blood spots and urine but can be normal (2). Unfortunately, we have no possibility to assess urinary adenosine. We could only assume that the result would be normal.

To confirm the final diagnosis, molecular analysis of *ADK* is essential (2–7). Based on our case report, we would like to highlight that ADK deficiency should be added to the list of genes involved in cholestatic liver disorders and liver cirrhosis.

### Mitochondrial respiratory chain defect in ADK deficiency

Due to the coexistence of liver disease and extrahepatic (neurological) manifestation, the mitochondrial RC defect was considered in our patient. It is well known that mitochondrial RC deficiencies may present as liver disease with cholestasis and/or liver failure, especially within the first months of life (14, 15). The presence of additional extrahepatic manifestations raises the suspicion of RC defects.

Becker et al. have recently reported for the first time the results of enzymatic studies of respiratory chain in liver samples of two patients with ADK deficiency (6). A decreased activity of I, II, V and I, II, IV complexes were found, respectively. In our patient, we found a decrease of mitochondrial RC complex II and III activity and increased activity of citrate synthase. We would like to highlight that our paper is the second report on mitochondrial dysfunction in ADK deficiency.

In the liver of Adk-deficient rats, levels of ATP were found reduced, which could impair RC functionality and exacerbate mitochondrial damage (16).

### Methionine restriction diet

According to recommendations for treatment of inherited methylation disorders, a low methionine diet should be considered as a therapeutic option in ADK deficiency (17). However, a dietary therapy has a limited effect (if at all) at the neurological phenotype, whereas it has a good impact on the hepatic phenotype (2–7). Methionine-restricted diet was not necessary in our patient given the fact that has been transplanted and has good liver function.

## Data Availability

The original contributions presented in the study are included in the article/Supplementary Material, further inquiries can be directed to the corresponding author.
